# miR-487b mitigates chronic heart failure through inhibition of the IL-33/ST2 signaling pathway

**DOI:** 10.18632/oncotarget.18393

**Published:** 2017-06-07

**Authors:** En-Wei Wang, Xu-Sheng Jia, Chang-Wu Ruan, Zhi-Ru Ge

**Affiliations:** ^1^ Department of Cardiac Surgery, Linyi People's Hospital, Linyi 276003, China; ^2^ Department of Cardiology, Gongli Hospital Affiliated to Second Military Medical University, Shanghai 200135, China

**Keywords:** miR-487b, IL-33, ST2, chronic heart failure, signaling pathway

## Abstract

We investigated the effects of microRNA-587b (miR-487b) in a rat model of chronic heart failure (CHF). Wistar rats were assigned to 10 groups (n=8 per group). Expression of interleukin-33 (IL-33), somatostatin 2 (ST2), IL-6, and TNF-α was higher in the CHF group than the control group. In the CHF, negative control (NC) for si-IL-33, NC for miR-487b mimic, NC for miR-487b inhibitor, and miR-487b inhibitor + si IL-33 groups, as compared to the blank and sham groups: steroid binding protein (SBP), D binding protein (DBP), left ventricular systolic pressure (LVSP), ± dp/dt_max_, and superoxide dismutase (SOD) were all lower; myocardial fibrosis, MDA, left ventricular end-diastolic pressure (LVEDP), myocardial apoptosis rate, IL-6, and TNF-α were all higher; levels of IL-33 and ST2 mRNA and protein were higher; and levels of miR-487b were lower. Levels of IL-33 and ST2 mRNA and protein were lower, and SBP, DBP, LVSP, ± dp/dt_max_, and SOD were higher in the miR-487b mimic and si-IL-33 groups than the CHF group. Expression of miR-487b was increased in the miR-487b mimic group, and expression of IL-33 and ST2 were increased and expression of miR-487b was decreased in the miR-487b inhibitor group. MiR-487b reduces apoptosis, inflammatory responses, and fibrosis in CHF by suppressing IL-33 through inhibition the IL-33/ST2 signaling pathway.

## INTRODUCTION

Heart failure (HF) is an abnormality of cardiac function or structure that causes the heart to deliver oxygen with increased filling pressures or fail to deliver oxygen at a rate required for metabolizing tissues, regardless of normal filling pressures [1]. As a progressive syndrome, chronic heart failure (CHF) is a major cause of disability and death on a global scale. CHF can decrease patients’ quality of life and increase economic pressure on the health care system and increase the lifetime risk of CHF. HF occurs in approximately 20% of patients older than 40 years [2, 3]. HF patients often have symptoms such as fatigue, breathlessness and ankle swelling, and they show signs such as pulmonary crackles, displaced apex beat, and elevated jugular venous pressure [1]. An aging population and the westernization of dietary habits account for the prevalence of CHF. An elevated resting heart rate is an overlooked risk factor in CHF [4–6]. Although progress has been made in the control of cardiovascular diseases, the incidence and prevalence of CHF continue to increase [7]. Thus new therapies are needed.

Endogenous small, noncoding miRNAs can stimulate gene expression via acceleration of mRNA activation or prevent translation through imperfect sequence-specific interaction with the 30-untranslated region of mRNAs, among which miR-487b is encoded by the 14q32.31 locus [8, 9]. Studies have suggested that miRNAs can be a new biomarker for CHF [10, 11]. The newly established member of the IL-1 (interleukin-1) family, interleukin-33 (IL-33), is expressed in a variety of cell types after proinflammatory stimulation and is released by cell lysis. Thus it is potentially useful for treating a variety of diseases [12]. Serum levels of IL-33 are expressed more frequently in CHF patients and it is positively correlated with markers of CHF severity [13]. As a member of the IL-1 receptor family, somatostatin 2 (ST2) was recognized as an orphan receptor in 1989, and clinical observations have associated ST2 with a range of diseases, including asthma, septic shock, pulmonary fibrosis, and rheumatoid arthritis, as well as collagen vascular diseases [14]. Ky et al [15] reported that ST2 is an efficient marker in CHF. The IL-33/ST2 pathway is correlated with the severity of HF clinical course and is a factor in the decline of cardiac function in HF patients [16]. In this context, we explored new therapeutic approaches for CHF through discussing miR-487b suppression of IL-33 to inhibit the IL-33/ST2 signaling pathway in that disease.

## RESULTS

### Comparisons of expressions of IL-33, sST2, miR-487b, IL-6, and TNF-α between the CHF group and the control group

**Figure 1:**
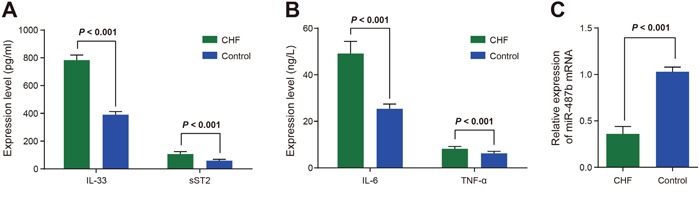
Comparisons of expressions of IL-33, sST2 **(A)** IL-6, and TNF-α **(B)** and miR-487b **(C)** between the CHF and control groups. CHF: chronic heart failure; IL-33: interleukin-33; ST2: somatostatin 2; TNF-a: tumor necrosis factor-α; miR-487b: microRNA-487b.

The results showed that the expressions of IL-33 and sST2 in the CHF group were higher than those in the control group (*P* < 0.05 for both; Figure 1A). Compared with the control group, the expressions of IL-6 and TNF-α were increased in the CHF group (*P* < 0.05 for both; Figure 1B), and that of miR-487b decreased in the CHF group (*P* < 0.05; Figure 1C).

### Relation between IL-33/ST2 signaling pathway-associated proteins and clinicopathological characteristics of CHF patients

The results showed that age and gender had no effects on the expression of IL-33/ST2 signaling pathway-associated proteins (*P* > 0.05). Also, there were no significant differences in IL-33 and sST2 expression in patients with DCM, HHD, and CHD (*P* > 0.05 for all), as well as in IL-33 expression in patients with different cardiac function classes (*P* > 0.05 for all). The expression of sST2 in patients in cardiac function class IV was higher than that in patients in class II and class III (*P* < 0.01 for all). The expression of sST2 in patients in class III was higher than that in patients in class II (*P* < 0.01; Table 1).

**Table 1:** Relationship between IL-33/ST2 signaling pathway-related proteins and clinicopathological characteristics (pg/ml)

Group		n	IL-33	sST2
Gender	Male	51	784.44 ± 38.11	107.35 ± 17.09
	Female	38	783.20 ± 33.81	106.09 ± 17.92
Age	< 45	8	797.92 ± 45.64	113.77 ± 20.87
	≥ 45	81	782.53 ± 35.11	106.12 ± 16.97
Pathological type	DCM	7	799.44 ± 45.76	95.88 ± 16.99
	Hypertension heart disease	38	789.60 ± 40.65	110.50 ± 19.94
	Coronary heart disease	44	776.52 ± 28.90	105.36 ± 14.17
Class	Class II	37	776.42 ± 37.00	92.19 ± 12.82
	Class III	28	791.81 ± 29.02	110.22 ± 5.87*
	Class IV	24	786.25 ± 41.16	125.37 ± 11.75*#

* refers to compare with class II, P < 0.05; # refers to compare with class III, P < 0.05. DCM: dilated cardiomyopathy; IL-33/ST2: interleukin-33/somatostatin 2; IL-33: interleukin-33; sST2: somatostatin 2.

### Mir-487b detection in myocardial tissue of rat

**Figure 2:**
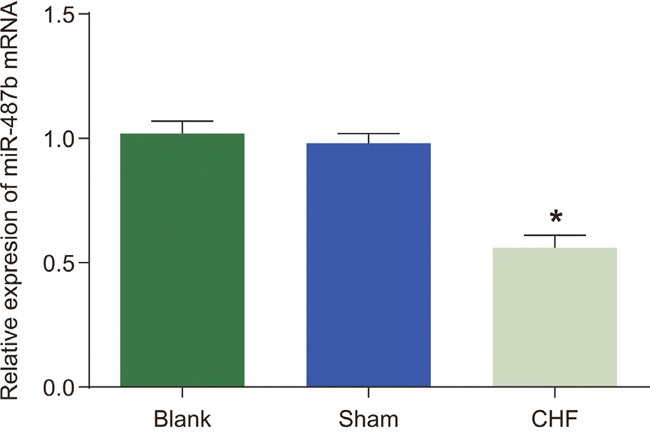
Effects of miR-487b expression in myocardial tissues of the blank, sham, and CHF groups CHF: chronic heart failure; miR-487b: microRNA-487b. Asterisk (*) indicates *P* < 0.05.

qRT-PCR was performed to detect the expression of mir-487b in myocardial tissue of rat in each group, which demonstrated that the expression of miR-487b decreased in the CHF group compared with the blank and sham groups (*P* < 0.05, for both; Figure 2).

### Comparisons of cardiac function of rats among eight groups

Results in Table 2 show a decrease in SBP, DBP, LVSP, and ± dp/dt_max_ but an increase in LVEDP in the CHF group compared with the blank and sham groups (*P* < 0.05 for all). Compared with the CHF group, there was an increase in SBP, DBP, LVSP, and ± dp/dt_max_ but a decrease in LVEDP in the miR-487b mimic group and the si IL-33 group (*P* < 0.05 for all) and decline in SBP, DBP, LVSP, and ± dp/dt_max_ in the miR-487b inhibitor group but an increase in LVEDP (*P* < 0.05 for all). There were no significant differences in cardiac function in the blank, sham, CHF, NC of miR-487b mimic, NC of miR-487b inhibitor, NC of si IL-33, and miR-487b inhibitor + si IL-33 groups (*P* > 0.05 for all). In addition, the HR in each group was not significantly different (*P* > 0.05 for all).

**Table 2:** Cardiac functions of rats in eight groups

Group	Blank	Sham	CHF	miR-487b mimic	NC of miR-487b mimic	miR-487b inhibitor	NC of miR-487b inhibitor	miR-487b inhibitor + si IL-33	si IL-33	NC of si IL-33
HR(time/min)	334 ± 20.85	353 ± 26.61	345 ± 23.22	341 ± 21.94	352 ± 23.13	355 ± 24.31	354 ± 21.31	347 ± 20.92	359 ± 29.70	353 ± 22.11
SBP(mmHg)	214.52 ± 17.26	205.39 ± 18.47	106.49 ± 10.26*	141.47 ± 17.96#	109.45 ± 8.91*	80.28 ± 4.97*#	106.54 ± 8.87*	110.30 ± 10.49*	137.71 ± 12.34#	108.24 ± 9.84*
DBP(mmHg)	181.35 ± 17.69	166.71 ± 14.03	100.21 ± 10.15*	137.28 ± 12.09#	101.19 ± 7.46*	70.81 ± 6.76*#	97.36 ± 8.23*	105.47 ± 9.03*	132.28 ± 15.92#	98.36 ± 7.19*
LVSP(mmHg)	256.21 ± 26.31	242.34 ± 22.93	162.47 ± 16.93*	199.77 ± 20.16#	158.32 ± 14.78*	130.71 ± 16.12*#	156.12 ± 14.42*	165.66 ± 16.05*	197.54 ± 21.73#	155.32 ± 15.76*
LVEDP(mmHg)	26.17 ± 3.92	28.46 ± 5.28	84.62 ± 5.97*	54.15 ± 5.87#	86.19 ± 7.16*	113.26 ± 14.30*#	88.38 ± 8.23*	85.61 ± 8.19*	57.18 ± 5.16#	82.38 ± 8.43*
+dp/dtmax(mmHg/s)	5449.26 ± 682.42	5339.22 ± 396.27	3986.24 ± 425.17*	4887.36 ± 486.41#	4170.53 ± 422.16*	3017.28 ± 338.19*#	4165.62 ± 431.79*	3799.63 ± 425.80*	4763.19 ± 547.23#	4173.62 ± 426.12*
-dp/dtmax(mmHg/s)	5531.47 ± 497.54	5428.36 ± 319.40	3392.48 ± 431.59*	4517.10 ± 536.37#	3484.91 ± 421.76*	2594.07 ± 318.71*#	3481.25 ± 437.69*	3401.54 ± 435.27*	4320.94 ± 559.82#	3482.25 ± 402.01*

* refers to compare with the blank group, P < 0.05; # refers to compare with the CHF group, P < 0.05. CHF: chronic heart failure; HR: heart rate; SBP: systolic blood pressure; DBP: diastolic blood pressure; LVSP: left ventricular systolic pressure; LVEDP: left ventricular end diastolic pressure; miR-487b: microRNA-487b; NC: negative control.

### Comparisons of heart morphology of rats among eight groups

**Figure 3:**
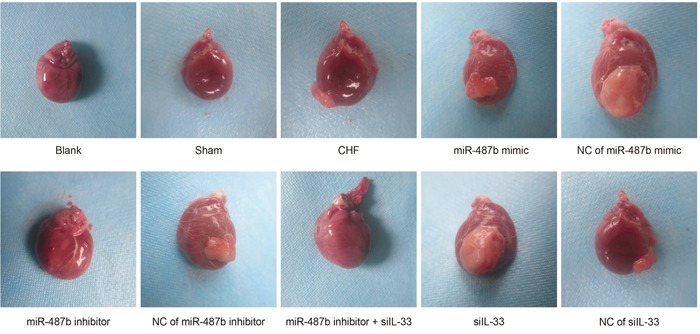
Morphology in myocardial tissues of eight groups

In the blank and sham groups, the rat hearts showed the normal sizes, shapes, and light red color. When the ventricle was incised, it showed normal size with RVWT and without ventricular aneurysm. Compared with the sham group, the heart volume was increased with changed geometry, thinner myocardium, and pale infarct, and most of the ventricles had ventricular aneurysm and the ventricular chamber was enlarged when the heart was incised in the CHF group. In the miR-487b mimic and si IL-33 groups, the heart had enlarged volume and maintained the normal geometry in dark red with pale infarct compared with the sham group. The infarct size decreased and the ventricle had less ventricular aneurysm and decreased dilation compared with the CHF group. Compared with the sham groups, rats in the miR-487b inhibitor group had differences, including increased heart volume, obvious inflammation, changed geometry, dark red heart, numerous pale infarcts, enlarged infarct size, increased ventricular aneurysm, and aggravated ventricle amplification (Figure 3). As shown in Tables 3 and 4, the HW/BW and LVW/BW were higher in the CHF, NC of miR-487b mimic, NC of miR-487b inhibitor, NC of si-IL-33, and miR-487b inhibitor + si IL-33 groups than those in the blank and sham groups (*P* < 0.05 for all). Compared with the CHF group, HW/BW and LVHW/BW were decreased in the miR-487b mimic and the si IL-33 groups (*P* < 0.05 for all), but increased in the miR-487b inhibitor group (*P* < 0.05 for both).

**Table 3:** Hemodynamic index in eight groups

Group	LVPWs(m)	LVPWd(m)	IVSs(mm)	IVSd(mm)	LVEF(mm)
Blank	0.27 ± 0.04	0.19 ± 0.03	0.28 ± 0.02	0.17 ± 0.04	89.24 ± 0.98
Sham	0.28 ± 0.05	0.19 ± 0.02	0.28 ± 0.04	0.17 ± 0.02	90.01 ± 1.24
CHF	0.44 ± 0.06*	0.32 ± 0.04*	0.43 ± 0.04*	0.30 ± 0.04*	61.54 ± 1.45*
miR-487b mimic	0.31 ± 0.02#	0.23 ± 0.04#	0.32 ± 0.03#	0.20 ± 0.02#	88.77 ± 1.29#
NC of miR-487b mimic	0.42 ± 0.02*	0.33 ± 0.03*	0.41 ± 0.03*	0.33 ± 0.05*	63.23 ± 0.87*
miR-487b inhibitor	0.52 ± 0.06*#	0.43 ± 0.07*#	0.53 ± 0.04*#	0.43 ± 0.03*#	49.87 ± 0.69*#
NC of miR-487b inhibitor	0.41 ± 0.03*	0.32 ± 0.04*	0.42 ± 0.03*	0.34 ± 0.03*	63.09 ± 1.01*
miR-487b inhibitor+si IL-33	0.42 ± 0.03*	0.31 ± 0.03*	0.41 ± 0.06*	0.32 ± 0.02*	62.98 ± 1.51*
Si-IL-33	0.33 ± 0.01#	0.24 ± 0.03#	0.34 ± 0.04#	0.20 ± 0.03#	87.99 ± 1.91#
NC of si IL-33	0.43 ± 0.05*	0.31 ± 0.04*	0.41 ± 0.05*	0.30 ± 0.05*	64.68 ± 1.63*

* refers to compare with the blank group, P < 0.05; # refers to compare with the CHF group, P < 0.05. LVPWs: left ventricular posterior wall thickness; LVPWd: left ventricular posterior wall thickness end-diastole; IVSs: systolic interventricular septum thickness; IVSd: interventricular septum thickness at end diastole; LVEF: left ventricular ejection fraction; CHF: chronic heart failure; miR-487b: microRNA-487b; NC: negative control.

**Table 4:** Comparisons of HW/BW and LVW/BW of rats among eight groups

Group	HW/BW	LVW/BW
Blank	3.05 ± 0.27	2.29 ± 0.23
Sham	3.46 ± 0.21	2.33 ± 0.19
CHF	4.64 ± 0.38*	3.41 ± 0.30*
miR-487b mimicNC of miR-487b mimic	3.39 ± 0.27#4.61 ± 0.28*	2.36 ± 0.16#3.43 ± 0.18*
miR-487b inhibitorNC of miR-487b inhibitor	5.33 ± 0.49*#4.67 ± 0.34*	4.59 ± 0.43*#3.18 ± 0.31*
miR-487b inhibitor+si IL-33	4.69 ± 0.41*	3.47 ± 0.32*
si IL-33NC of si IL-33	3.42 ± 0.22#4.59 ± 0.33*	2.30 ± 0.21#3.46 ± 0.27*

* refers to compare with the blank group, P < 0.05; # refers to compare with the CHF group, P < 0.05. CHF: chronic heart failure; HW: heart weight; BW: body weight; LVW: left ventricular weight; miR-487b: microRNA-487b; NC: negative control.

### Effects of myocardial fibrosis of rats in eight groups

**Figure 4:**
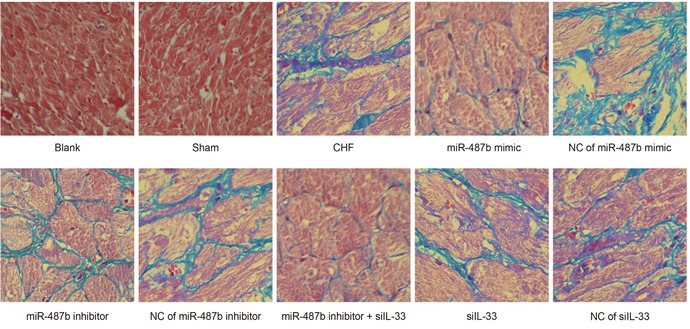
The myocardial tissues of rats detected by Masson staining among eight groups (× 200)

Under a microscope, the green collagen fibers, the pink myocardial cells and the blue nucleus were observed. In the sham and blank groups, the myocardial cells were morphologically normal and arranged neatly without or with rare collagen fibers. In the CHF, NC of miR-487b mimic, NC of miR-487b inhibitor, NC of si-IL-33, and miR-487b inhibitor + si IL-33 groups, the myocardial cells had hypertrophy and many green collagen fibers, were decreased in number, and were disorderly arranged in the intercellular space. In the si IL-33 and miR-487b mimic groups, the myocardial cells were dominant, and a few branching collagen fibers alternated with the myocardial cells. In the CHF group, the myocardial cells were increased and the green collagen fibers in intercellular space were decreased. Inflammatory hypertrophy decreased and myocardial cells were disordered in the miR-487b inhibitor group, but the green collagen fibers were increased compared with the CHF group (Figure 4).

### Apoptosis in myocardial cells of rats among eight groups

**Figure 5:**
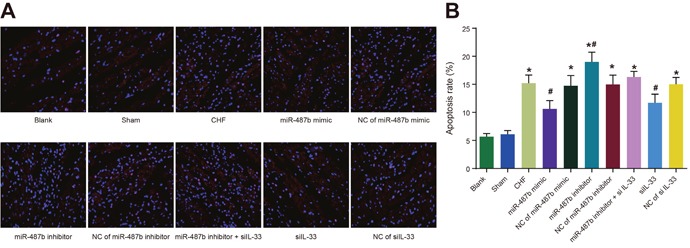
Comparisons of apoptosis of myocardial cells **(A)** Apoptosis of myocardial cells in eight groups detected by TUNEL staining (× 400). **(B)** Levels of apoptosis of myocardial cells in 10 groups. TUNEL: terminal deoxynucleotidyl transferase dUTP nick end labeling; CHF: chronic heart failure. Asterisk (*) indicates comparison with the control group, *P* < 0.001. Hash symbol (#) indicates to comparison with the CHF group, *P* < 0.005.

A small number of apoptotic myocardial cells were in the blank and sham groups. The number of apoptotic myocardial cells in each group were more than in the blank and sham groups (*P* < 0.05 for all), and the number in the si IL-33 and miR-487b mimic groups were less than that in the CHF group, but more than that in the blank and sham groups (*P* < 0.05 for all). The number in the miR-487b inhibitor group increased compared with the CHF group (*P* < 0.05), and there were no significant differences in the NC of miR-487b mimic, NC of miR-487b inhibitor, NC of si IL-33, and miR-487b inhibitor + si IL-33, and CHF groups (*P* > 0.05 for all) (Figure 5).

### IL-6 and TNF-α expressions in myocardial tissues of rats among eight groups

**Figure 6:**
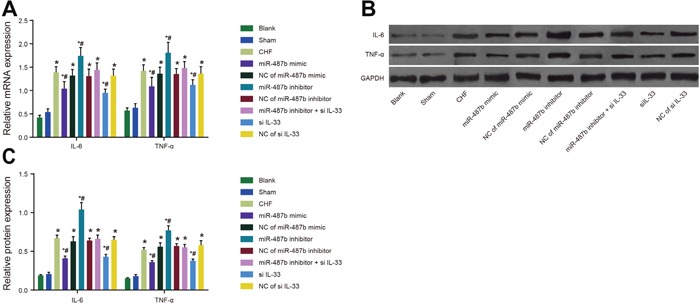
Expressions of IL-6 and TNF-α in rat myocardial tissues in each group detected by qRT-PCR and Western blotting **(A)** The expressions of IL-6 and TNF-α mRNA in rat myocardial tissues of each group. **(B-C)** The protein expressions of IL-6 and TNF-α in rat myocardial tissues of each group. IL-6: interleukin-6; TNF-α: tumor necrosis factor-α; CHF: chronic heart failure. Asterisk (*) indicates to comparison with the blank group, *P* < 0.05. Hash symbol (#) indicates comparison with the CHF group, *P* < 0.05.

The qRT-PCR and Western blotting were performed to detect mRNA and protein expressions of IL-6 and TNF-α in rat myocardial tissues. Expression in each group increased compared with expression in the blank and sham groups (*P* < 0.05 for all), and expression in the si IL-33 and miR-487b mimic groups were lower than in the CHF group but higher than in the blank and sham groups (*P* < 0.05 for all), and expression in the miR-487b inhibitor group increased compared with the CHF group (*P* < 0.05 for both). There were no differences among the mRNA and protein expressions of IL-6 and TNF-α in the NC of miR-487b mimic, NC of miR-487b inhibitor, NC of si IL-33, miR-487b inhibitor + si IL-33, and CHF groups (*P* > 0.05 for all) (Figure 6).

### SOD activity and MDA content in myocardial tissues of rats among eight groups

Compared with the blank and sham groups, MDA content of myocardial tissues in each model group increased, whereas SOD activity decreased (*P* < 0.05 for all). SOD activity and MDA in the si IL-33 group and the miR-487b mimic group were less than in the CHF group, but more than in the blank and sham groups. SOD activity increased but less than in the blank and sham groups (*P* < 0.05 for all). Compared with the CHF group, the miR-487b inhibitor group had an increase in MDA content but a decrease in SOD activity. MDA content and SOD activity were not significantly different in the NC of miR-487b mimic, NC of miR-487b inhibitor, NC of si IL-33, miR-487b inhibitor + si IL-33, and CHF groups (*P* > 0.05 for all) (Table 5).

**Table 5:** SOD activity and MDA content in rat myocardial tissues among eight groups

Group	SOD (U/ml)	MDA (mol/ml)
Blank	349.82 ± 26.74	7.84 ± 0.62
Sham	347.17 ± 25.38	7.71 ± 0.59
CHF	236.34 ± 22.19*	16.82 ± 1.48*
miR-487b inhibitor	184.26 ± 17.33*#	19.26 ± 1.54*#
NC of miR-487b inhibitor	237.24 ± 9.65*	16.87 ± 1.75*
miR-487b inhibitor + si IL-33	233.50 ± 22.07*	16.94 ± 1.53*
NC of si IL-33	239.48 ± 22.35*	16.53 ± 1.28*

* refers to compare with the blank group, P < 0.05; # refers to compare with the CHF group, P < 0.05. SOD: superoxide dismutase; MDA: malondialdehyde; CHF: chronic heart failure; miR-487b: microRNA-487b; NC: negative control; IL-33: interleukin-33.

### Comparisons of miR-487b expression and mRNA expressions of IL-33/ST2 signaling pathway-associated proteins of rats among eight groups

**Figure 7:**
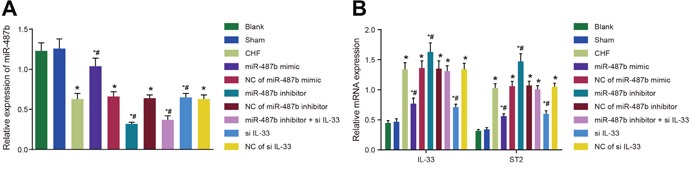
The mRNA and expressions of miR-487b **(A)** and IL-33/ST2 signaling pathway-associated proteins **(B)** in rat myocardial tissues of eight groups. miR-487b: microRNA-487b; IL-33/ST2: interleukin-33/somatostatin 2; CHF: chronic heart failure. Asterisk (*) indicates comparison with the blank group, *P* < 0.05. Hash symbol (#) indicates comparison with the CHF group, *P* < 0.05.

The results of qRT-PCR showed that when compared with the blank and sham groups, IL-33 and ST2 mRNA expression increased, whereas miR-487b expression decreased in the CHF, NC of miR-487b mimic, NC of miR-487b inhibitor, NC of si-IL-33, and miR-487b inhibitor + si IL-33 groups (*P* < 0.05 for all). Compared with the CHF group, IL-33 and ST2 mRNA expression decreased in the miR-487b mimic and si IL-33 groups, whereas miR-487b expression in the miR-487b mimic group was higher than that in the CHF group (*P* < 0.05 for all), and IL-33 and ST2 mRNA expression increased, whereas miR-487b expression decreased in the miR-487b inhibitor group (*P* < 0.05 for all) (Figure 7).

### Comparisons of protein expressions of IL-33/ST2 signaling pathway-associated proteins of rats among eight groups

**Figure 8:**
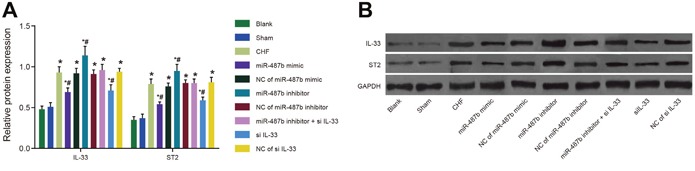
The expressions of IL-33/ST2 signaling pathway-associated proteins in rat myocardial tissues among eight groups **(A)** Relative protein expression of IL-33 and ST2. **(B)** western blotting bands. IL-33/ST2: interleukin-33/somatostatin 2; CHF: chronic heart failure. Asterisk (*) indicates comparison with the blank group, *P* < 0.05. Hash symbol (#) indicates comparison with the CHF group, *P* < 0.05.

As shown in Figure 8, compared with the blank and sham groups, ST2 and IL-33 protein expression increased in the CHF, NC of miR-487b mimic, NC of miR-487b inhibitor, NC of si IL-33, and miR-487b inhibitor + si IL-33 groups (*P* < 0.05 for all) but decreased in the miR-487b mimic group and the si IL-33 group (*P* < 0.05 for all). Protein expression was higher than in the miR-487b inhibitor group compared with the CHF group (*P* < 0.05 for all).

### A correlation was found between miR-487b and IL-33

**Figure 9:**
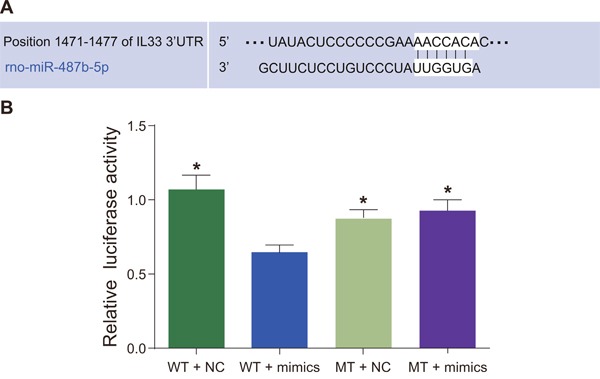
The verification of the relation between miR-487b and IL-33 **(A)** Bioinformatics software for predicting the relation between miR-487b and IL-33; **(B)** a dual luciferase reporter system for verifying the relation between miR-487b and IL-33. WT: wild type; MT: mutant; miR-487b: microRNA-487b; IL-33: interleukin-33. Asterisk (*) indicates comparison with the WT + mimics, *P* < 0.05.

Bioinformatics software (http://www.targetscan.org) was used to predict a correlation between miR-487b and IL-33 (Figure 9A). IL-33-3′UTR-WT plasmid and miR-487b stimulated plasmid were co-transfected in 293T cells, which showed that compared with the control group (IL-33-3′UTR-WT + NC), the mean value of the luciferase activity ratio of firefly to renilla (Y/H) decreased (*P* < 0.05), whereas the mean value of Y/H for co-transfection of IL-33-3′UTR-MUT + miR-487b simulated plasmid was not significantly different compared with the control group (*P* > 0.05; Figure 9B).

## DISCUSSION

This study investigated the effects of miR-487b activation of IL-33 through inhibition of the IL-33/ST2 signaling pathway in CHF, and we found that miR-487b suppresses the apoptosis, the inflammatory reaction of myocarditis, and myocardial fibrosis.

Results imply that miR-487b decreases IL-6 and TNF-α expression, and both increased SOD and blocked MDA were identified in the CHF group. The 14q32 miRNA cluster belongs to one of the largest polycistronic clusters and consists of 54 miRNAs in humans and 61 in mice, and miR-487b is a member of this cluster [17]. MiRNAs, including miR-487b, can function as posttranscriptional inhibitors, and in the context of inflammation they inhibit the production of cytokines such as IL-6 and TNF-α by repressing gene transcription, preventing translation, and destabilizing mRNA [18]. Moreover, downregulation of miR-487b increases invasion, tumorigenicity and proliferation of cells. Therefore, the expression of miR-486b inhibits cell growth in lung cancer *in vivo* and *in vitro* [19]. SOD is a protective enzyme against oxidative stress, which is a factor in CHF. SOD reduces oxidative stress and protects cardiomyocytes from apoptosis and fibrosis, so it is rational to assume that the higher the SOD expression, the less severe the CHF [20, 21]. Zhang et al [13] reported that IL-33 reduces eSOD (a form of SOD) production and IL-33 increases in CHF, so eSOD decreases in CHF. Oxidative stress is correlated with elevated levels of lipid peroxides, including MDA, so in the CHF group, MDA is upregulated [22].

We found that the miR-487b mimic group showed downregulated IL-33 and ST2 as well as upregulated miR-487b, and less cell apoptosis, inflammatory responses of myocarditis, and fibrosis were observed in this group, suggesting miR-487b alleviates CHF through inhibition of the IL-33 and ST2 signaling pathway. HF is one of the most serious types of cardiovascular diseases, including atherosclerosis, cardiac fibrosis, and hypertrophy, which were all correlated with IL-33 and ST2 because IL-33 binding to ST2 not only leads to maladaptive pro-hypertrophic but also causes pro-fibrotic responses to mechanical stretch and stress (although IL-33 treatment reduces hypertrophy and fibrosis, sST2 prevents anti-hypertrophic effects of IL-33) [23, 24]. The IL-33/ST2 signaling pathway is an intercellular signaling system that is a factor in fibrosis, antigen–allergen response, and autoimmunity [25]. By suppressing caspase-3 activity and enhancing the expression of the inhibitor of apoptosis (IAP) family of proteins, IL-33 reduces fibrosis and cardiomyocyte apoptosis and improves ventricular function, whereas the elevated sST2 expression inhibits IL-33 function, but miR-487b promotes the protective response of IL-33 in the heart because it binds to the IL-33 3’-untranslated region to activate IL-33 transcripts [24, 26]. IL-33/ST2 signaling is a protective pathway in different cardiovascular diseases, and IL-33 induces immune responses during atherosclerosis [27]. The present results demonstrate that IL-33/ST2 lead to carrageenan-induced congenital inflammation, which depends on the activation of recruited leukocytes and resident tissue cells [28]. Yamazumi et al [32] reported that miR-487b was negatively correlated with the expression IL-33, and because expression of IL-33 and ST2 showed the same changing direction in CHF patients, it is plausible that miR-487b also inhibits ST2 [13, 29].

Our data demonstrated that miR-487b ameliorates cell apoptosis, inflammatory reaction of myocarditis, and fibrosis through inhibiting the IL-33/ST2 pathway by suppressing IL-33, providing a novel therapy for CHF. However, the clinical value of this study remains to be tapped and verified.

## MATERIALS AND METHODS

### Ethics statement

This study has been approved by the Ethics Committee of Linyi People's Hospital, and all patients signed the consent form. All procedures in this study were in strict accordance with the guidelines and principles of the Declaration of Helsinki.

### Study object

A total of 89 patients with CHF (mean age 64.75 ± 14.45 years), including 51 males and 38 females, were admitted at Linyi People's Hospital from February 2013 to December 2014 as the CHF group. Patients were all in compliance with the diagnostic criteria of CHF issued by the National Institute for Health and Clinical Excellence in 2010 [30]. Exclusion criteria were malignant tumors and conditions such as active infection, systemic inflammation, autoimmune disease, and asthma. The control group comprised 70 healthy patients consisting of 37 males and 33 females with a mean age of 66.64 ± 11.36 years. The full clinical data of all study subjects were recorded. According to New York Heart Association heart function classification [31], all CHF patients were classified as class II (37 patients), class III (28 patients), or class IV (24 patients). Based on etiology, all CHF patients were divided into dilated cardiomyopathy (7 patients), hypertension heart disease (38 patients), and coronary heart disease (44 patients).

### Enzyme-linked immune sorbent assay

Peripheral venous blood was collected from CHF patients as soon as they were admitted into Linyi People's Hospital. Tubes containing the blood were reserved at 45- to 60-degree angles at a temperature of 4° C for 1 hour, followed by 5 minutes of centrifugation at 3000 rpm/min to obtain a supernatant that was kept at −20° C to detect concentrations of IL-33, somatostatin 2 (sST2), IL-6, and tumor necrosis factor-α (TNF-α) in the supernatant by use of an ELISA kit (West Tang Bio-tech Co., Ltd., Shanghai, China). Every ELISA experiment was repeated three times to determine the average value.

### Construction of CHF rat model

A total of 80 Wistar rats (40 males and 40 females) aged 8 weeks with body weight of 250 ± 30 g were purchased from Shanghai Jia Ke Biotechnology Co. Ltd., China. All rats were fed standard laboratory animal fodder and had free access to tap water at 25° C in 12-hour simulation of day and night 1 week in advance. The rats were divided into 2 groups: the blank group (treated with nothing) and the sham group (coronary artery occlusion without ligation operation). Ligation of the anterior descending artery was performed in 64 rats [32]. A solution of 3% pentobarbital sodium (P3761, Sigma-Aldrich Chemical Company, St. Louis, MO, USA) was intraperitoneally injected into rats to induce anesthesia, and then the rats’ four limbs were fixed to an experiment table, followed by insertion of needle electrodes into subcutaneous tissue to observe and record the standard limb lead II. A rubber ball the size of a rat's head and neck was used to connect the rats’ heads with the respirator for artificial respiration (respiration frequency, 55 time/min; tidal volume, 18-25 mL). After removal of the chest hair and disinfection, a 2-cm longitudinal skin incision was cut along left mid-clavicular line. Later, subcutaneous tissue and muscle were separated passively layer-by-layer via the fourth or fifth intercostal space, followed by pouch suture for reservation. With oxygen inhalation, the third intercostal space was separated to open the chest, and the heart was exteriorized by lightly pressing the right profile. After the descending branch of the left anterior descending (LAD) coronary artery was ligated between the arterial cone and auricular appendix, the rat heart was put back into the chest as soon as possible, followed by discharging the gas in the chest. Then the chest was closed and spontaneous breathing restored as the artificial respiration was stopped. Rats in the sham group underwent the same procedure, except no ligation was performed, and the normal group had no treatment. After surgery, the rats were given penicillin 10,000 U·time/rat for 3 days to prevent infection. The left ventricular ejection fraction (LVEF) was reduced by 45% by use of an ultrasonic cardiogram to determine the success of the CHF rat model.

### *In vivo* transfection and grouping

DNA oligonucleotides of overexpressed and low-expressed RNA of miR-487b were synthesized to activate the sequence that effectively promotes miR-487b expression, and later DNA oligonucleotides were made into double-stranded DNA, followed by connection and transformation with pGCSIL-GFP vector digested by Age I and EcoR I for screening positive clones by polymerase chain reaction (PCR), and plasmid was extracted, digested, and sequenced. We synthesized the overexpressed subject of miR-487b (miR-487b mimic) and the inhibitor of miR-487b (miR-487b inhibitor) and suppressed the expression subject of IL33 (si IL-33). For each rat models, 200 μg of nucleic acid, 400 μL of pGCSIL-GFP Entranster™-*in vivo* (Engreen Biosystem Co. Ltd., Beijing, China), and 10% glucose solution were mixed to a total of 1.5 mL of solution, followed by stewing for 15 minutes at room temperature and injection via the rat's tail to construct a transfection model after a one-week adaptive experiment. The rats were divided into 10 groups (8 rats of each group): the blank group (treated with nothing), the sham group (coronary artery occlusion without ligation operation), the CHF group (construction of the CHF rat model), the miR-487b mimic group (tail vein injection of miR-487b mimic after one-week adaptive experiment), miR-487b inhibitor group (tail vein injection of miR-487b inhibitor), the negative control (NC) group (including NC of si-IL-33, NC of miR-487b mimic, and NC of miR-487b inhibitor; tail vein injection of nonsense sequence), the si IL-33 group (tail vein injection of IL-33 siRNA sequence), and the miR-487b inhibitor + si IL-33 group (transfection of miR-487b inhibitor and IL-33 siRNA sequence). After the successful construction of the CHF model, caudal vein injection was conducted in the rats to transfect a sequence in the miR-487b mimic, control, NC of si-IL-33, NC of miR-487b mimic, NC of miR-487b inhibitor, si-IL-33, and miR-487b inhibitor + si-IL-33 groups, repeated every 3 days. All the rats had detected heart function after transfection for 4 weeks, and then the heart was exteriorized.

### Determination of cardiac function

Rats were anesthetized with 3% pentobarbital sodium (30 mg/kg) via intraperitoneal injection under electrocardiograph monitoring. The heparin-filled catheter was inserted into the left ventricle via the right common carotid artery to connect the pressure transducer and the AP-621 G carrier amplifier. An RM-6000 type 8-path physiological recorder was used to record left ventricular systolic pressure (LVSP) and left ventricular end diastolic pressure (LVEDP). Then the LVSP electrical signal was input into a differentiator to calculate the maximal rate of increase and decrease of ventricular pressure (± dp/dt_max_). Diastolic blood pressure (DBP), systolic blood pressure (SBP), and heart rate (HR) were recorded via femoral artery intubation.

### Hemodynamic parameters detection

Rats were anesthetized by inhalation of 3% isoflurane (0.2 L/min medical air) once at 4-weeks and 8-weeks after construction of the rat model. After anesthesia, rats were fixed in a supine position to preserve the skin. The left ventricular posterior wall thickness in systole (LVPWs), left ventricular posterior wall in diastole (LVPWd), intraventricular septal end systole (IVSs), intraventricular septal end diastole (IVSd), left ventricular ejection fraction (LVEF) of rat at the long axis of the left ventricle (LV), and abdominal aorta were detected by use of ultrasonic diagnostic equipment

### Heart morphology detection

After surgery, the hearts were removed and lavaged repeatedly with cold normal saline for 4 weeks and dried with filter paper so the heart morphology could be observed. Later, heart weight (HW) and left ventricular weight (LVW) were determined to calculate the heart hypertrophy index (HW/body weight [BW]) and left ventricular hypertrophy index (LVW/BW). Then the heart cavities were incised to observe the ventricular geometric shapes and wall thicknesses to determine whether the rats had ventricular aneurysms.

### Myocardial fibrosis detection

Masson staining was performed to analyze myocardial fibrosis at the right ventricle, and the steps were as follows: rat myocardial tissues at the right ventricle were fixed with 4% paraformaldehyde, embedded in paraffin, and later cut into 5-μm slices. After dewaxing and washing, myocardium slices were stained with hematoxylin at 60° C for 30 to 60 seconds, washed with running water, differentiated with 1% hydrochloric acid alcohol, and turned back to blue with bluing solution for 5 to 10 seconds. Then myocardium slices were stained with Harris hematoxylin for 3 minutes, washed with running water, differentiated with 1% hydrochloric acid alcohol, and washed with running water again, followed by 1-minute immersion in warm water (approximately 50° C) and running-water washing. Later, myocardium slices were stained with ponceau acid fuchsin for 3 minutes, washed with distilled water, differentiated with 1% phosphorus aluminum acid, and re-stained with 2% aniline blue for 1 minute, followed by 95% ethanol dehydration and mounting in neutral reins.

### Terminal deoxynucleotidyl transferase dUTP nick end labeling assay

Myocardial tissues at the right ventricle were washed with phosphate buffer solution (PBS) and fixed with neutral formaldehyde solution at 4° C for 24 hours for later preparation of paraffin section. After conventional dewaxing, the slices were used for cell apoptosis detection according to the manufacturer's instructions (Jiancheng Bioengineering Co., Nanjing, China). Five views were selected randomly from each slice to calculate the numbers of apoptotic cells and total numbers of cells under light microscope. Apoptosis rate = numbers of positive apoptotic cells/numbers of total cells × 100%. The terminal deoxynucleotidyl transferase dUTP nick end labeling (TUNEL) assay was repeated three times to determine the mean value.

### Detection of superoxide dismutase activity and malondialdehyde content

The right ventricle was separated from peeled rat heart, cleaned by pre-cooled normal saline, dried by filter paper, and weighed, and ice physiological saline (weight/volume:1/9) was added to make 10% homogenate, followed by a centrifugation at 3000 rpm/min for 15 minutes to collect a supernatant. The activity of superoxide dismutase (SOD) was detected by use of a xanthine oxidase technique as directed by a kit purchased from Shanghai Solarbio Bioscience & Technology Co. Ltd., China, and the MDA content was determined by thiobarbituric acid (TBA) method as directed by a kit purchased from Shanghai Solarbio Bioscience & Technology Co. Ltd., China. All procedures were in strict accordance with manufacturer's instructions.

### Quantitative real-time polymerase chain reaction

Quantitative real-time polymerase chain reaction (qRT-PCR) was performed to detect the expression of miR-487b, IL-33, ST2, IL-6, and TNF-α mRNA in right ventricular myocardial tissues in each group. The Trizol (Invitrogen Inc., California, USA) method was conducted to extract the total RNA. Ultraviolet analysis and formaldehyde gel electrophoresis were used to verify the high-quality RNA. A total of 1 μg of RNA was extracted for reverse transcription by avian myeloblastosis virus (AMV) reverse transcriptase to synthesize cDNA. Additionally, β-actin was used as an internal reference. PCR primers were designed and synthesized by Shanghai Invitrogen Co. Ltd., China (Tables 6 and 7), with β-actin as an internal reference. PCR reaction conditions were pre-denaturation at 94° C for 5 minutes, 40 cycles of amplification (denaturation at 96° C for 30 seconds, annealing at 60° C for 40 seconds, and extension at 72° C for 1 minute), and final extension at 72° C for 10 minutes. The electrophoresis analysis of PCR products was carried out on agar gel, and PCR results were analyzed by Opticon Monitor 3 software (Bio-Rad Inc., Hercules, CA, USA). The threshold was manually determined, and the lowest point of parallel rise in each logarithm amplification curve was selected as the Ct value (cycle threshold) of each reaction tube, and all data were analyzed by the 2^-ΔΔCt^ method. In this method, 2^-ΔΔCt^ refers to multiple proportions of the target gene expression in the experiment group and the control group. The formula is ΔΔCt = [Ct (target gene) – Ct (reference gene)]_experiment group_ – [Ct (target gene) – Ct (reference gene)]_control group_. This experiment was repeated three times, and the average value was used.

**Table 6:** Primer sequence of individuals

Gene	Primer sequence
miR-487b	F: TGCGGAATCGTACAGGGTCATCCA
	R: CCAGTGCAGGGTCCCAGGT
U6	F: TGCGGGTGCTCGCTTCCGCACC
	R: CCAGTGCAGGGTCCGAGGT

miR-487b: microRNA-487b; F: forward; R: reverse.

**Table 7:** Primer sequence of rats

Gene	Primer sequence
miR-487b	F:AGGTAAGAGTAGAGCAGC
	R:AATCGTACAGGGTCATC
U6	F: CTCGCTTCGGCAGCACA
	R: AACGCTTCACGAATTTGCGT
IL-33	F:CACCCCTCAAATGAATCAGG
	R:GGAGCTCCACAGAGTGTTCC
ST2	F:AATCGTCCTGGGGTCT
	R:GGCGGCTTTTTATGTA
IL-6	F:GAAAACACCAGGGTCAGCAT
	R:CAGCCACTGGTTTTTCTGCT
TNF-α	F:CTCCTACCCGAACAAGGTCA
	R:CGGTCACCCTTCTCCAACT
β-actin	F:GAATCCACTGGCGTCTTCAC
	R: CGTTGCTGACAATCTTGAGAGA

F: forward; R: reverse; miR-487b: microRNA-487b; IL-33: interleukin-33; ST2: somatostatin 2; TNF-α: tumor necrosis factor-α.

### Western blotting

Myocardial cell proteins of the right ventricle were extracted to detect the protein concentration, according to BCA kit instructions (Boster Bioengineering Co. Ltd., Wuhan, China). Cell were added to the electrophoresis chamber, and then added with a loading buffer, boiled at 95° C for 10 minutes, and a 30-μg sample was put into each well of a 10% polyacrylamide gel to conduct electrophoresis to isolate the proteins. Electrophoresis voltage was changed from 80 v to 120 v, and the wet-transfer method was performed to transfer protein to a polyvinylidene fluoride (PVDF) membrane at 100 mv for 45 to 70 minutes. After being sealed in 5% bull serum albumin (BSA) at room temperature for 1 hour, samples were incubated with rabbit-anti-mouse primary antibodies IL-33 (1:1000) and ST2 (1:1000) (Cell Signaling Technology Inc., Danvers, MA, USA), anti-mouse primary antibody IL-6 (1:5000) and TNF-α (1:5000) (Bioss Bio-technology Co. Ltd., Beijing, China), and anti-mouse primary antibody β-actin (1:3000) (Becton, Dickinson and Company, Franklin Lakes, NJ, USA) at 4° C overnight. After being washed by Tris Buffered Saline with Tween (TBST) three times (5 minutes × 3), samples were incubated with goat-anti-rabbit secondary antibody (Miao Tong Bio-technology Co. Ltd., Shanghai, China) at room temperature for 1 hour and washed with TBST three times (5 minutes × 3), followed by film development via addition of chemiluminescence reagent. β-actin was used as an internal reference. Bio-rad Gel Dol EZ imager (Bio-Rad Inc., Hercules, CA, USA) was used to develop the film, and Image J software analyzed the gray value. The experiment was repeated three times, and the average value was used.

### Dual luciferase reporter gene system

Bioinformatics software (http://www.targetscan.org) was used to predict the relation between miR-487b and IL-33, and a dual luciferase reporter system was used to verify the gene. According to the instructions in an enzyme activity assay kit (Promega Corp., Madison, WI, USA), a 96-well plate was washed by PBS, and later 100 μL of phospholamban (PLB) solution was added to each well, followed by a 15-minute oscillation at low velocity to collect cell lysis buffer. After 20 μL of cell lysis buffer was mixed with 100 μL of luciferase detection agent II, a luminometer (TD20/20; Turner Designs, Sunnyvale, CA, USA) was used to read the collecting activity value of ranilla luciferase. Then, 100 μL of 1 × Stop & Glo solution was added and evenly mixed to collect the activity value of ranilla luciferase. The reporter gene expression was a ratio of firefly luciferase to renilla luciferase.

### Statistical analysis

The statistical analysis was performed by SPPSS 21.0 software (SPSS Inc., Chicago, IL, USA). Measurement data were expressed as mean ± standard deviation (SD). Pairwise comparison was analyzed by the least significant difference (LSD) method, and comparisons among groups were analyzed by the one-way ANOVA method, and the *t*-test was performed to compare measurement data compliance with normal distribution between two groups. *P* < 0.05 was considered statistically significant.
